# A Rare Case of Complete Heart Block in a Young Patient

**DOI:** 10.1155/2018/1493121

**Published:** 2018-06-06

**Authors:** Zakaria Hindi, Yousef Hindi, Rami Batarseh

**Affiliations:** ^1^Internal Medicine Department, Texas Tech University Health Sciences Center, Permian Basin, Odessa, TX, USA; ^2^Cardiology Department, University of Texas Health Science Center at San Antonio, San Antonio, TX, USA

## Abstract

**Introduction:**

Complete heart block (CHB) is considered as one of the dangerous rhythms since it can progress to lethal arrhythmias such as ventricular tachycardia. It can be congenital or acquired. Patients may present with frequent palpitations, presyncope, dyspnea, or chest pain but also may remain asymptomatic. Extensive work-up should be conducted to exclude secondary causes such as infections, cardiac ischemia or myopathies, autoimmune diseases, or endocrinological diseases. In our paper, we would like to present a case of CHB in the setting of aortic abdominal thrombus that nearly occluded both renal arteries. The CHB in this case is thought to be caused by hypertensive cardiomyopathy due to ongoing uncontrolled hypertension, which is caused by bilateral renal artery stenosis.

**Case Presentation:**

A 31-year-old male with history of active smoking was incidentally found to have high blood pressure, bradycardia, and CHB on electrocardiogram. The patient was admitted to a cardiology ward and extensive work-up revealed hypokinesia of the left ventricle with low ejection fraction and left ventricle concentric hypertrophy, large abdominal aortic thrombus with bilateral renal artery stenosis, and evidence of arterial collateral connections, which suggest chronicity. The patient then was placed on four antihypertensive medications but eventually, he underwent bilateral renal artery stenting and insertion of permanent pacemaker for his CHB. The patient's blood pressure then was under control with only one medication, and subsequent CT angiogram showed no evidence of stenosis of both renal arteries.

**Conclusion:**

Uncontrolled hypertension can lead to hypertensive cardiomyopathy, which in turn can cause conduction abnormalities such as CHB. Although hypertension can be secondary to a treatable underlying cause, permanent pacemaker is essential to treat CHB.

## 1. Introduction

Complete heart block (CHB) is considered as one of the dangerous rhythms since it can progress to lethal arrhythmias such as ventricular tachycardia. It can be congenital or secondary to infections, cardiac ischemia or myopathies, autoimmune diseases, or endocrinological diseases that require extensive work-up to be ruled out [1]. Out of secondary causes, hypertrophic obstructive cardiomyopathy and infiltrative myopathies such as sarcoidosis and amyloidosis are considered as potential causes for CHB [2].

Taking hypertensive heart disease into consideration, the condition is quite similar to hypertrophic cardiomyopathy. In that, both conditions have similar findings on physical exam and echocardiogram. They can be differentiated however on the basis of strain rate imaging [3]. Although the establishment of relation between hypertrophic cardiomyopathy and CHB has been reported before [4], yet the association between hypertensive cardiomyopathy and CHB has never been reported. Hence, we would like to report a case of CHB in a patient with bilateral renal artery stenosis, nonpreserved nonischemic hypertensive cardiomyopathy, and uncontrolled hypertension. We believe that this case would merit further investigation regarding the relation between hypertensive cardiomyopathy and CHB.

## 2. Case Presentation

**Figure 1:**
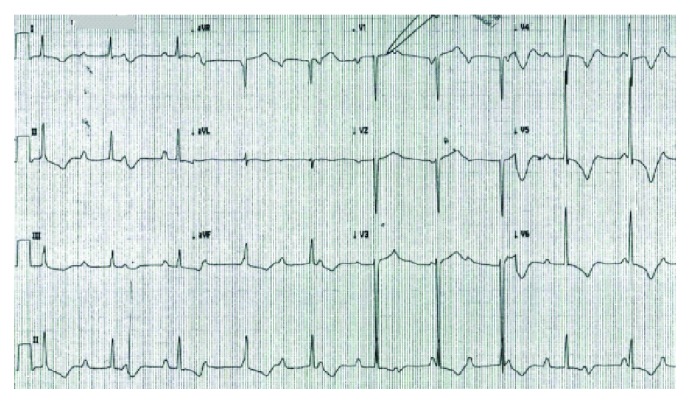
EKG showing complete heart block with T wave inversion in inferiolateral leads and LVH strain.

A 30-year-old male with history of active smoking (1 pack per day for 10 years) and external hemorrhoids came to the preop anesthesia clinic for anesthesia evaluation fitness and was found to have high blood pressure (BP) (234/144). He was referred immediately to the emergency room (ER) for BP control. In the ER, BP was 221/125 and heart rate (HR) was 50 beats/minute. Routine electrocardiogram (EKG) showed 3rd-degree heart block (TDHB) and left ventricular hypertrophy (LVH) with strain pattern (Figure 1). He denied chest pain, palpitation, dyspnea, dizziness, or syncope. The patient was started on antihypertensive medication for BP control and was admitted to the cardiology ward for evaluation and management of complete heart block. Further physical exam revealed absent arterial pulses except the left radial pulse which was weak. BP was significantly different between both upper limbs and between upper and lower limbs (right upper limb 126/86 and lower limb 85/54, left upper limb 145/85 and lower limb 75/50). His initial blood work showed mild renal impairment.

**Figure 2:**
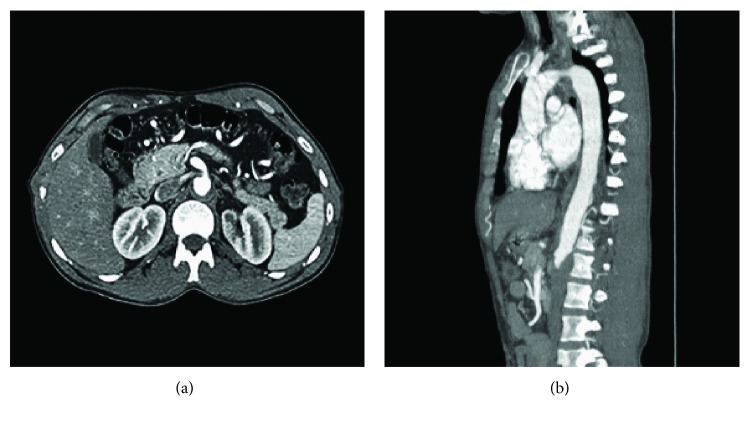
CT angiogram of the abdomen. (a) Bilateral stenosis and near occlusion of renal arteries. (b) Abdominal aortic thrombus that extends to the level of renal arteries.

**Figure 3:**
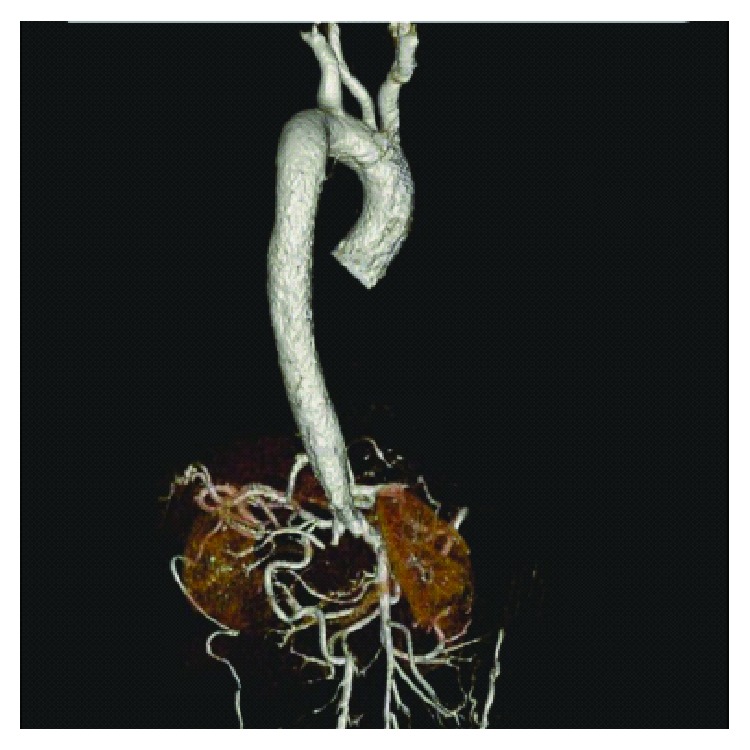
Aortogram extraction scan. It shows collateral arteries around renal arteries.

Computerized tomography (CT) thoracic aortogram was done to rule out coarctation of the aorta, which was normal; CT coronary angiogram showed no evidence of coronary artery disease (CAD). Magnetic resonance imaging (MRI) of the heart was normal as well. Transthoracic echocardiogram (TTE) showed moderate hypokinesia of the left ventricle (LV), ejection fraction (EF) 35–40%, grade 2/4 diastolic dysfunction, and moderate concentric LVH. Holter monitoring did not reveal any pauses. Ultrasound/Doppler of the kidneys showed increased parenchymal echogenicity with poorly defined corticomedullary junction impressive of renal parenchymal disease. CT abdominal aortogram showed large thrombus seen in the abdominal aorta starting at the level of renal arteries completely occluding the aorta and common iliac arteries with no blood flow seen beyond the renal artery level up to the aortic bifurcation; moderate to severe stenosis is noted at the origin of both renal arteries because of thrombus (Figures 2(a) and 2(b)). Multiple abdominal collaterals are seen with multiple collaterals in the anterior and lateral abdominal wall and paraspinal collaterals (Figure 3). Extensive blood work-up including thyroid function test and autoimmune and thrombophilia work-up was all unremarkable. No cause of aortic thrombosis and TDHB was identified.

Initial recommendation of the vascular surgeon was to follow up in the clinic with no intervention as it is a chronic process, and the patient was asymptomatic. Since the patient had uncontrolled hypertension despite being on maximum doses of four antihypertensive medications, eventually he underwent percutaneous stenting of bilateral renal arteries which was followed by an improvement in the BP and renal function and reduction in doses of antihypertensive medications. The patient also underwent permanent pacemaker insertion for TDHB. The patient was also placed on warfarin and was advised to see the vascular surgeon after 3 months. Unfortunately, the patient did not follow up.

## 3. Discussion

This young patient had accidental findings of asymptomatic CHB, uncontrolled HTN with renal artery stenosis, and complete thrombosis of the abdominal aorta. After reviewing the literature, we could not find a unifying diagnosis for the patient's condition. The closest condition to our patient's presentation is Leriche's that is described as an aortoiliac disease, which partially explains the patient's thrombotic events [5]. Yet, Leriche's syndrome has no known association with CHB. We believe that the patient had initially bilateral renal artery stenosis which was a consequence of the chronic abdominal aortic thrombus as there were collateral arteries. The uncontrolled HTN which resulted from untreated bilateral renal artery stenosis led to hypertensive heart disease that was evident by EKG and echocardiogram findings.

Hypertensive heart disease (HHD) is a group of abnormalities that comprises LVH and systolic/diastolic dysfunction. HHD in general is considered as a risk factor for atrial and ventricular arrhythmias such as atrial fibrillation, supraventricular tachycardia, and ventricular tachycardia. Bradyarrhythmias, however, are mainly caused by drug side effects (such as beta blockers) when treating HTN [6–8]. The pathogenesis of arrhythmias in HHD arises mainly from LVH and left ventricular dysfunction [9]. Both may be associated with CHB if ischemic cardiomyopathy develops [10]. In our patient, ischemic cardiomyopathy was ruled out by coronary angiogram. This can indicate that HHD may cause atrioventricular block without developing ischemia.

The management of our patient was based on controlling HTN sufficiently. Our target blood pressure was below 130/80 mmHg. Despite medicating the patient with maximum doses of four different antihypertensive agents (which fits the definition of resistant HTN [11]), his blood pressure was not controlled. The stenting of both renal arteries was done as per American Heart Association recommendations [11]. As for CHB, the treatment of choice is permanent pacemaker regardless of the symptoms [12, 13].

## 4. Conclusion

It should be emphasized that CHB may be a rare consequence in HHD which requires permanent pacemaker regardless of cardiomyopathy stage or severity of symptoms. Moreover, the relation between CHB and HHD should be investigated.
